# Delta-8-THC association with psychosis: A case report with literature review

**DOI:** 10.3389/fpsyt.2023.1103123

**Published:** 2023-02-20

**Authors:** Chelsea R. Miller, Bradley G. Burk, Rachel E. Fargason, Badari Birur

**Affiliations:** ^1^Department of Psychiatry and Behavioral Neurobiology, University of Alabama at Birmingham, Birmingham, AL, United States; ^2^Department of Pharmacy, University of Alabama at Birmingham, Birmingham, AL, United States

**Keywords:** delta-8-tetrahydrocannabinol, delta-8-THC, delta-9-tetrahydrocannabinol, psychosis, *Cannabis sativa*, case report

## Abstract

**Background:**

Cannabis (Δ^9^-THC) is the most commonly consumed illicit drug. The Agricultural Improvement Act of 2018 removed hemp, a strain of *Cannabis sativa*, as a controlled substance. This law allowed the plant to be processed into its components, which contain <0.3% Δ^9^-THC. As a result, delta-8-tetrahydrocannabinol (Δ^8^-THC), a federally unregulated substance, grew in popularity in 2020. Δ^8^-THC is readily available in most gas stations or head shops and may be considered harmless by patients. However, an increasing number of patients admitted for psychiatric hospitalization report use, with limited literature on the effects.

**Case presentations:**

This case report describes three individual cases of patients who required admission to a university psychiatric hospital after the regular use solely of Δ^8^-THC. All three patients developed psychotic and paranoid symptoms concurrently with the use of Δ^8^-THC, with a severity exceeding their previous historical presentations. The presenting psychotic symptoms were also atypical for all three patients. New-onset violence and visual hallucinations were noted in two of the patients, one patient with no previous psychiatric history and one patient while on a therapeutic dose of his antipsychotic. In the third case, a new onset of bizarre, fixed delusions of puppies dissolving in the bathtub developed.

**Conclusion:**

This report adds to the limited body of evidence on Δ^8^-THC documenting a temporal association between Δ^8^-THC use and the development of psychotic symptoms. A strong body of research already correlates the continued use of Δ^9^-THC with psychosis, and Δ^8^-THC acts at the same CB_1_ and CB_2_ receptors as Δ^9^-THC. Therefore, it is hypothesized that Δ^8^-THC may have similar adverse psychiatric effects as Δ^9^-THC. These conclusions are not without speculation, due to the need for self or collateral-reporting of Δ^8^-THC use as urine drug screening cannot distinguish Δ^8^-THC from Δ^9^-THC, and the patients' symptoms could be explained by medication non-adherence and primary psychotic disorders. However, physicians should be encouraged to gather a specific history of Δ^8^-THC use and treat patients with Δ^8^-THC-related intoxication and symptoms.

## 1. Introduction

Cannabis is the most frequently consumed illicit drug (1, 2), with over 16 million Americans reporting current use (3). Cultivated from the *Cannabis sativa* plant, the cannabinoid analog responsible for the euphoric “high” of cannabis is delta-9-tetrahydrocannabinol (delta-9-THC, Δ^9^-THC). A concentration of more than 0.3% of Δ^9^-THC is considered a Schedule I controlled substance under the Controlled Substance Act of 1970 (4) at the federal level and is a Schedule I controlled substance (carrying the highest legal penalties) in 32 states. Schedule I substances are classified as having no medical use and are of high potential for abuse (5). Other cannabinoids of the *C. sativa* plant, such as cannabidiol (CBD), do not produce intoxicating effects and are federally recognized as a Schedule V substance by the Drug Enforcement Administration (DEA) with a Δ^9^-THC concentration of <0.1% (6) and recognized as legal by the Food and Drug Administration (FDA) when marketed as cosmetic products only (7). CBD is considered illegal if companies try to make health claims about CBD in products as only one product, Epidiolex (an antiepileptic), is FDA-approved for medical treatment (7).

When the Agricultural Improvement Act of 2018, also known as the Farm Bill, removed hemp, a chemovar of *C. sativa* called *C. sativa* L., as a controlled substance (8), it created a significant legal loophole for the production of cannabis-derived products. The Farm Bill allowed for *C. sativa* L (hemp) to be processed and sold into its individual components (such as CBD, hemp seed oil, and fibers for textiles), as long as they contain <0.3% Δ^9^-THC (9). Following the passing of this law, the legal sale of a non-controlled psychoactive substance, delta-8-tetrahydrocannabinol (delta-8-THC, Δ^8^-THC), emerged, gaining popularity in late 2020 (10).

**Figure 1:**
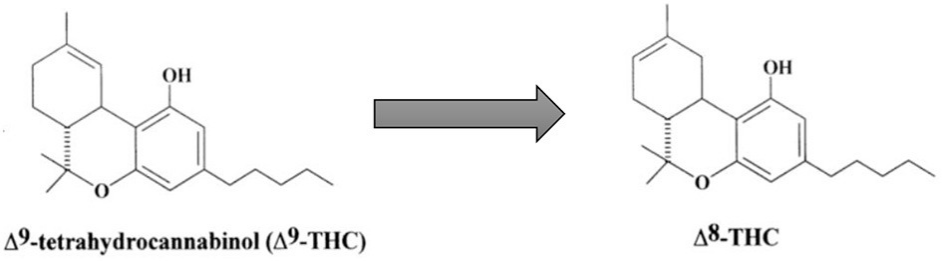
Conversion of Δ^9^-THC to Δ^8^-THC. Adapted with permission from Elsevier, Mechoulam, R. and L. Hanuš (2002). “Cannabidiol: an overview of some chemical and pharmacological aspects. Part I: chemical aspects.” Chemistry and physics of lipids 121(1-2): 35-43. https://doi.org/10.1016/S0009-3084(02)00144-5.

Delta-8-THC is an isomer of delta-9-THC, synthetically formed by the cyclization of CBD to move the double bond from the 9th position to the 8th position within the cyclohexane ring (11), as shown in Figure 1. This changes the molecule to an officially non-controlled substance because it is no longer structurally Δ^9^-THC, despite being a psychoactive substance of the same molecular weight. However, the DEA stated in 2021, in an Interim Final Rule on Δ^8^-THC, that any Δ^8^-THC derived from chemical conversion or synthetic methods is illegal (4). Since Δ^8^-THC occurs in low quantities naturally in the *C. sativa* L. (hemp) plant (12), the majority of Δ^8^-THC in commerce is synthesized from CBD oil from these hemp plants (10). The DEA essentially contradicts its regulation as it indicates synthetic cannabinoids to be manmade, non-organic, and synthesized in a lab setting (4, 13), a ruling which is also confirmed by the FDA which defines synthetic material to be substances not found in nature (4). These discrepancies provoked a letter to be written by the DEA to the Alabama Board of Pharmacy to clarify that any Δ^8^-THC product would not be considered a Schedule I substance if it was produced from hemp, and contains <0.3% Δ^9^-THC (13).

Unfortunately, without regulation, Δ^8^-THC products can have variability in their concentration. An independent study that tested 51 Δ^8^-THC products found 76% of them contained greater than the legal limit allowed of Δ^9^-THC (14). Furthermore, little is known about the other byproducts created from the cyclization process (15) or other isomers of THC created by the virtue of synthetization. One toxicology study noted that Δ^8^-THC products contain heavy metals such as lead, mercury, and tin (16), which can have detrimental health effects on consumers. Furthermore, residual solvents from the synthesis of Δ^8^-THC could also have potential toxicology that is unknown. The Centers for Disease Control and Prevention (CDC) had 660 exposures to Δ^8^-THC reported in the first 6 months of 2021, of which 18% required hospitalization (17). The CDC warns about the sedative effects of intoxication with Δ^8^-THC, and also warns users who rely on packaging that reporting of Δ^9^-THC concentration may underestimate the total THC concentration and result in the psychoactive potential of the substance being consumed (17).

Despite ongoing uncertainty around Δ^8^-THC legal status and safety, there were 22.3 million internet searches for Δ^8^-THC in the first 8 months of 2021 (18), reflecting the increasing public interest in Δ^8^-THC. It is likely medical providers are seeing more cases of Δ^8^-THC intoxication but documented that reporting of this presentation is limited by the inability of a standard urine drug screen to distinguish between Δ^8^-THC and Δ^9^-THC (19). In forensic cases that required distinction, liquid chromatography with high-resolution mass spectrometry was required to distinguish between Δ^8^-THC and Δ^9^-THC (19), after screening urine immunochemical analysis showed Δ^8^-THC to have >100% cross-reaction to the Δ^9^-THC antibodies (20).

Thus far, there are two published case reports documenting Δ^8^-THC intoxication. The first case describes acute encephalopathy in a pediatric patient following accidental ingestion of Δ^8^-THC gummies (21). The second case report documents a 23-year-old male and a 35-year-old female who self-reported Δ^8^-THC use before presenting with delusions and suicidal ideation that was persistent and resistant to conventional treatment (22). This manuscript also presents three additional cases, in which the chief complaint was psychosis in self-reported Δ^8^-THC users who did not report using other substances, summarized in Table 1.

**Table 1:** Summary of cases.

**Age**	**Sex**	**Prior psychiatric diagnosis**	**Type of Δ8-THC**	**Presenting symptoms**	**Treatment**	**Length of stay in days**
21	Male	None	Vaping	Violence Psychosis-VH and AH Paranoia	Paliperidone Palmitate	32
23	Male	Schizophrenia intellectual disability	Gummies	Violence Psychosis-VH and AH Paranoia	Paliperidone Palmitate	19
39	Male	Schizoaffective disorder traumatic brain injury borderline intellectual functioning	Smoking delta-8 flowers	Paranoia Delusions	Aripiprazole	4

VH, visual hallucinations; AH, auditory hallucinations.

## 2. Case reports

### 2.1. Case 1

Mr. A, a 21-year-old male with no prior psychiatric history, was brought to the emergency room by police after being found wandering outside talking to himself. On arrival, the patient was afebrile at 98.1°F, with a heart rate (HR) of 64, respiratory rate (RR) of 18, and blood pressure (BP) of 136/96. The patient's complete blood count (CBC) and complete metabolic panel (CMP) were unremarkable, and a urine drug screen (UDS) was not obtained due to the patient being uncooperative with sample collection. Computed tomography (CT) of the head was negative for acute intracranial findings. On psychiatric evaluation, the patient was noted to have psychosis, as evidenced by responding to internal stimuli, laughing inappropriately, disorganization of thought process, reports of significant paranoia, hearing voices, and visual hallucinations. His behavior displayed disorganization with psychomotor agitation and suspiciousness of medical providers. Patient A endorsed vaping Δ^8^-THC but denied all other drug use. The patient was admitted to inpatient psychiatry.

The family reported that there was no family history of psychiatric illness. They also reported that patient A had a decline in behavior over the 8 months preceding his hospitalization but had no formal psychiatric diagnosis or psychological disturbance before this time. He displayed aggressive, violent outbursts and once got into a physical altercation with the family's landlord, causing them to be evicted from their home. Due to the patient's unpredictable behavior, the family feared him and had not recently allowed him to live in their home.

On day 1 of admission, the patient was started on oral risperidone 0.5 mg twice daily to address continued signs of psychosis. The patient exhibited disorganization, illogical thought process, and auditory and visual hallucinations. On day 3, his risperidone was increased to 1 mg twice daily and switched to 2 mg nightly on day 5. On the 10th day of admission, the patient still had poor insight into his hospital presentation. He continued to appear confused and disorganized during the interview, with difficulties in attention and concentration, as well as signs of paranoia. His risperidone was increased to 3 mg nightly; however, the above symptoms persisted. On day 20, his risperidone was increased to 4 mg nightly. On day 22, it was suspected that the patient was cheeking his medication, and was switched to risperidone orally disintegrating tablet 4 mg. By day 28, the patient was no longer endorsing visual or auditory hallucinations, was no longer paranoid, and was participating in group therapy.

During the hospitalization, the treatment team learned that the patient started using Δ^8^-THC around the same time that the family reported a decline in his behavior. This is also when the patient endorsed auditory hallucinations; however, the patient was unable to give a clear history of whether he started Δ^8^-THC to try and prevent the voices or if the voices occurred after the Δ^8^-THC use. He was started on an intramuscular paliperidone long-acting injectable (LAI) on the 28th day of admission and an oral risperidone taper was begun.

Patient A was discharged on the 32nd day after admission on a regimen of intramuscular paliperidone LAI 156 mg every 4 weeks (its metabolite is the same as risperidone and can be given every 4 weeks for improved adherence). He was back to his baseline functioning at discharge and was no longer endorsing signs or symptoms of psychosis and had an organized thought process without emotional lability. He was encouraged to abstain from any cannabis products.

Patient A, unfortunately, did not follow-up for his scheduled outpatient appointment after discharge; however, he has not presented to our hospital's ER in the 7 months following discharge.

### 2.2. Case 2

Mr. B is a 23-year-old male with a past medical history of schizophrenia and intellectual disability who presented to the emergency department via EMS after having a violent outburst at home. He broke the windows out of his house and tried to “arrest” his mother, stealing her car keys and breaking her arm. The patient was currently treated with intramuscular aripiprazole LAI 400 mg every 4 weeks and was 3 weeks post-last injection. His other home medication was divalproex delayed-release (DR) tablets 500 mg twice daily. His last inpatient psychiatric admission was a year before his presentation to the hospital. On arrival to the emergency department, he was afebrile, with HR 100, RR 15, BP 132/86, and labs were notable for a valproic acid level <10 and standard UDS positive for only THC. CMP, liver function tests (LFTs), CBC, and urinalysis (UA) were unremarkable.

The patient's mother reported that he had never been violent before, but that she had seen a change in his behavior over the previous several weeks, where he was talking and laughing to himself more, was preoccupied, and had been destroying things in his house. Patient B had been having difficulty sleeping and was self-medicating with Δ^8^-THC gummies for several months, which his mother reported buying him. On psychiatric exam, the patient was significantly withdrawn, disorganized, paranoid, and responding to auditory and visual hallucinations. The patient was also unkempt and malodorous, seemingly having difficulty with self-care.

The patient was admitted to inpatient psychiatry for acute care. On his first day of admission, he was maintained on his home dose of oral divalproex 500 mg twice daily and started on 6 mg of oral paliperidone daily. His aripiprazole LAI was not continued. He was still exhibiting disorganization, had visual hallucinations he was responding to, and continued to have poor hygiene. On day 3, his paliperidone was increased to 9 mg daily, and by day 6, the patient was more organized and able to hold a full conversation with the treatment team. He showed insight by recognizing that he could have hurt his mother and expressed remorse for his actions. On day 13, his paliperidone was increased to 12 mg daily, as the patient was remaining in his room and continued to have poor hygiene. By day 15, the patient was attending group therapy in the milieu and was bathing appropriately. He was given his first loading dose of intramuscular paliperidone LAI on day 16 of hospitalization, and his oral paliperidone was decreased by 3 mg daily until discontinuation. He was discharged on day 19 on a regimen of oral divalproex DR 500 mg twice daily and intramuscular paliperidone LAI 234 mg every 4 weeks (its metabolite is the same as risperidone and can be given every 4 weeks for improved adherence). On the day of discharge, he was no longer responding to internal stimuli, was not paranoid, and was performing his activities of daily living.

Patient B has continued to follow-up with his outpatient provider and has done well on this regimen in the 8 months following inpatient treatment. He has abstained from Δ^8^-THC use and has not had further psychiatric emergencies requiring hospitalization.

### 2.3. Case 3

Mr. C is a 39-year-old male with a history of schizoaffective disorder, traumatic brain injury (TBI), and borderline intellectual functioning, who presented to the ER complaining that he felt unsafe at home. Patient C was historically prescribed oral aripiprazole 2 mg daily. On arrival, he was afebrile, with HR 68, RR 18, and BP 127/83. CBC, CMP, and UA were unremarkable. UDS was positive for only THC. On psychiatric evaluation, the patient reported paranoid and bizarre delusions that someone was dissolving puppies in his bathtub at home with various chemicals. He had tried to involve the local and federal authorities and was encouraged by his minister to come to the hospital. The patient was perseverative on delusions and exhibited disorganization in speech, and was endorsing low mood and energy, poor sleep, and feelings of emptiness. He was disheveled with limited hygiene. The patient reported smoking Δ^8^-THC daily for 8 months, and self-discontinuation of his aripiprazole 4 months prior.

Per the family reports, though the patient has a TBI, he is the primary caretaker for his mother who has chronic medical issues. The family reports that the patient behaves appropriately when on his medications and does not mention delusions or display confusion throughout the day.

On the second day of admission, patient C was started on oral aripiprazole 2 mg, after he initially refused medication. He began to gain some insight that some of his delusions might not be true. After he was increased to aripiprazole 5 mg daily on the 3rd day of admission, he showed less perseveration on the delusions and was more future-oriented thinking about the need to help his mother around the house. On the 4th day of admission, he was discharged with instructions to increase his aripiprazole to 10 mg daily at home and follow up closely with his outpatient psychiatric provider, as well as to abstain from Δ^8^-THC products.

Patient C did not show up for his 3-week outpatient follow-up appointment but has left messages at the nurses' line stating similar delusions from his presentation to the hospital. He has not presented to the hospital again in the 3 months following discharge.

## 3. Literature review and discussion

Cannabinoid receptors are G-protein coupled receptors of the endocannabinoid system, in which the CB_1_ receptor has been shown to propagate the psychotropic effects of Δ^9^-THC (23). From the research available comparing Δ^8^-THC to Δ^9^-THC, it appears that Δ^8^-THC acts at both the cannabinoid CB_1_ and CB_2_ receptors, like Δ^9^-THC (24), but with a weaker affinity at the CB_1_ receptor (11, 25). CB_1_ receptors are also implicated in pain and sensory perception, emotional regulation, attention and concentration, memory, and mood (26). Δ^8^-THC may have a similar effect on the CB_2_ receptor as Δ^9^-THC, but fewer data exist (11). CB_2_ receptors are thought to play a role in the inflammatory response (27). Research in mice has shown CB_2_ to be part of the biphasic reward response, where lower activation increases dopamine in the nucleus accumbens and high activation is aversive, decreasing dopamine (28). Route of administration may also play a role in the potency of Δ^8^-THC and Δ^9^-THC. Some studies suggest that oral ingestion of Δ^8^-THC or Δ^9^-THC may be more potent than other routes of administration (29, 30) due to the metabolite 11-OH-THC produced from first-pass metabolism. 11-OH-THC has been shown in rat models to have a higher binding affinity for CB_1_ receptors, which are associated with the perceived high (11, 31).

Despite Δ^8^-THC being a newer substance of mainstream interest, several limited qualitative studies exist that compared Δ^8^-THC to Δ^9^-THC. The first reported qualitative study of Δ^8^-THC in humans occurred in 1942 by Adams (30). Adams studied male subjects in prison and showed Δ^8^-THC had similar qualitative effects (anxiety, euphoria, disinhibition, loquaciousness, laughter, and drowsiness) on prisoners as Δ^9^-THC. In 1973, Hollister and Gillespie (32) had a limited study of six male subjects, who reported Δ^8^-THC to be approximately two-thirds as potent as Δ^9^-THC based on self-reported symptoms, peak effect, and subjective intensity. However, cannabis is more potent now than four decades ago (1), with estimates of current Δ^9^-THC concentrations up to 20% (3) vs. 0.35% in commonly confiscated marijuana in the 1970s (33). Thus, the similarities reported in early studies may be more pronounced than what might be seen in current comparative studies of Δ^8^-THC and Δ^9^-THC.

Studies of Δ^8^-THC since its 2020 popularity are limited. One survey showed that one in six Δ^9^-THC users report Δ^8^-THC use (34). A survey study by Kruger and Kruger compared Δ^8^-THC with Δ^9^-THC, where participants who use Δ^8^-THC reported similarities in euphoria between the substances, consistent with Adams, Hollister, and Gillespie, without the reported side effects of paranoia and sedation experienced with Δ^9^-THC (10). This study had a homogenous population that over-selected white male subjects with 30% of their study population living in New York State. The demographic clustering noted in this study could suggest specific demographics of the users of Δ^8^-THC. Interestingly, this study touted Δ^8^-THC to be a tool for diversion in line with the ideals of harm reduction due to Δ^8^-THC's non-controlled status. However, for reasons discussed previously, lack of controlled status of a substance does not constitute safety.

The U.S. Food and Drug Administration recognizes Δ^8^-THC as a psychoactive substance and has had increased reporting of adverse experiences in users of hallucinations, increased anxiety, confusion, and loss of consciousness (35). Given that previous studies have shown similar euphoric experiences in users, along with binding to CB_1_ and CB_2_ receptors, could mean that Δ^8^-THC acts similarly to Δ^9^-THC. There is a strong body of evidence that associates the use of Δ^9^-THC with psychosis (36–42) with the greatest risk stemming from early initiation (43), and frequent use of high-potency Δ^9^-THC (44, 45). There is continued debate on whether there is a causality between Δ^9^-THC use and the emergence of schizophrenia (39, 46–48). Genetic predisposition (44) may also lead users of cannabis to experience worsening psychotic-like experiences. Furthermore, long-term use of Δ^9^-THC can lead to neuropsychological impairment, including reduced executive functioning and difficulties with concentration and memory (2, 49) that may (50) or may not (51) be reversible with abstinence. Based on the current reported data from qualitative studies on Δ^8^-THC, and the new evidence in rat models of cannabinoid receptor bonding of Δ^8^-THC in comparison to Δ^9^-THC, there is a possibility that Δ^8^-THC could also predispose to, or bring out, psychosis and neuropsychological decline similarly to Δ^9^-THC. However, more research is needed to assess if the same risk factors for psychosis and Δ^9^-THC use (frequency, potency, and genetic predisposition) also apply to the risk of psychosis with the use of Δ^8^-THC.

For the patients presented in the cases, all three developed psychotic symptoms concurrently with self-reported Δ^8^-THC use. The first two patients experienced violent behavior that was uncharacteristic per family collateral. The first two patients also experienced auditory and visual hallucinations. It is the standard of care to screen for organic causes of psychosis when a patient presents with visual hallucinations, as visual hallucinations are thought to be half as prevalent as auditory hallucinations within the psychotic disorder spectrum (52, 53). It is possible that Δ^8^-THC contributed to the patient's experience of visual hallucinations. Δ^9^-THC is known to cause transient psychotomimetic experiences such as derealization, depersonalization, dissociation, hallucinations, and paranoia (38), which all of the patients in the cases described did, in some part, report. Given the similarity of Δ^8^-THC to Δ^9^-THC, it is conceivable that Δ^8^-THC elicited or contributed to the symptoms leading to these patients' presentation. However, the psychotic symptoms were not transient and improved only upon initiation of antipsychotics, hence we cannot rule out the influence of the agent on activating a latent or existing primary psychotic disorder.

Acute Δ^9^-THC use can cause the release of dopamine in the brain (28, 54, 55), which may explain why concurrent use reduces the efficacy of antipsychotics and may lead to relapse of psychosis and re-hospitalizations in patients with schizophrenia (56). Again, the similarities between Δ^8^-THC and Δ^9^-THC could suggest that Δ^8^-THC would have comparable effects, which may be illustrated with patient B. Although patient B was non-compliant with his divalproex, and experienced insomnia, he had active antipsychotic coverage by his LAI and yet relapsed with concerning and severe psychotic symptoms. It is within reason to think that the Δ^8^-THC gummies he had been using were contributory, especially considering patient B has not relapsed on a different LAI post-hospitalization while abstaining from Δ^8^-THC use.

Patient A had no previous psychiatric history, no family history of psychosis, and no history of prodromal symptoms, and started using Δ^8^-THC around the same time he started to experience psychotic symptoms. The precise timing of the onset of symptoms in relation to his Δ^8^-THC use is unclear; however, patient A had a prolonged hospitalization due to psychosis and surreptitiously discarded his antipsychotic medication. Once he started reliably receiving regular doses of his medication, his symptoms appeared to resolve quickly. Finally, patient C was not compliant with his aripiprazole at home but was on a potentially sub-therapeutic dose to treat his schizoaffective disorder (57). His symptoms resolved quickly with hospitalization, and the patient began to suspect his thoughts were incorrect even before re-starting his aripiprazole. As one would expect aripiprazole to take longer than 2 days to help reduce the severity of delusions (58), perhaps abstaining from Δ^8^-THC for several days aided in his thought clarity.

These conclusions from the aforementioned cases are speculative with concern to Δ^8^-THC, and all patients' symptoms could be explained by medication non-adherence and primary psychotic disorders. However, anecdotally, there has been an increase in the use of Δ^8^-THC within our patient population, and the large body of evidence on cannabis (Δ^9^-THC) association with psychosis and schizophrenia cannot be ignored. Furthermore, other cases exist documenting similar presentations of patients using Δ^8^-THC (21, 22). A recognized limitation of Δ^8^-THC reporting is that a UDS cannot distinguish Δ^8^-THC from Δ^9^-THC. Another limitation beyond the subjectivity of information obtained for this case report is that samples of the Δ^8^-THC were not collected for toxicology to know the concentration of Δ^8^-THC or Δ^9^-THC within the product. Broader studies on the use of Δ^8^-THC are also difficult, as no ICD-10 code exists for Δ^8^-THC. Further research is needed to study how the balance of activation between CB_1_ and CB_2_ receptors affects psychotomimetic experiences and how route determines pharmacokinetics, along with clinical data in human models to determine differences in response to Δ^8^-THC and Δ^9^-THC.

## 4. Conclusion

It is recommended that providers ask patients or family members specifically about Δ^8^-THC when approaching the social history of the psychiatric interview or medical history. Patients may deny all drug use since Δ^8^-THC is not a controlled substance, have a positive UDS for THC, and could present with symptoms as described earlier. By asking directly about Δ^8^-THC, providers can use ICD-10 code F12.92 to denote cannabis use, unspecified with intoxication, and report synthetic cannabinoid Δ^8^-THC as part of further comments under the diagnosis within the electronic medical record. Finally, this and other reports suggest that tighter regulation be placed on substances synthesized from hemp and that the federal government confirm their classification of synthesized substances through the DEA and FDA. This may help with legislation at the local and state levels, as well as regulation of delta-8 products, to ensure consistency and standard of product for the safety of the user.

## Data availability statement

The original contributions presented in the study are included in the article/supplementary material, further inquiries can be directed to the corresponding author.

## Ethics statement

Written informed consent was obtained from the individual(s) for the publication of any potentially identifiable images or data included in this article.

## Author contributions

CM, BrB, and BaB were involved in the assessment and medication management of the patients. CM wrote the first draft of the manuscript. BrB, BaB, and RF contributed to the preparation of the manuscript and have approved the final version of the manuscript. All authors contributed to the article and approved the submitted version.
